# Combination simvastatin and metformin induces G1-phase cell cycle arrest and Ripk1- and Ripk3-dependent necrosis in C4-2B osseous metastatic castration-resistant prostate cancer cells

**DOI:** 10.1038/cddis.2014.500

**Published:** 2014-11-20

**Authors:** M A Babcook, R M Sramkoski, H Fujioka, F Daneshgari, A Almasan, S Shukla, R R Nanavaty, S Gupta

**Affiliations:** 1 Department of Nutrition, Case Western Reserve University School of Medicine, Cleveland, OH 44106, USA; 2 Department of Urology, Case Western Reserve University School of Medicine & The Urology Institute, University Hospitals Case Medical Center, Cleveland, OH 44106, USA; 3 Cytometry & Imaging Microscopy Core Facility, Case Comprehensive Cancer Center, Cleveland, OH 44106, USA; 4 Electron Microscopy Core Facility and Center for Mitochondrial Disease, Case Western Reserve University School of Medicine, Cleveland, OH 44106, USA; 5 Department of Pharmacology, Case Western Reserve University School of Medicine, Cleveland, OH 44106, USA; 6 Department of Cancer Biology, Lerner Research Institute, Cleveland Clinic, Cleveland, OH 44195, USA; 7 Department of Radiation Oncology, Taussig Cancer Institute, Cleveland Clinic, Cleveland, OH 44195, USA; 8 Department of Biomedical Science, The Ohio State University, Columbus, OH 43210, USA; 9 Division of General Medical Sciences, Case Comprehensive Cancer Center, Cleveland, OH 44106, USA

## Abstract

Castration-resistant prostate cancer (CRPC) cells acquire resistance to chemotherapy and apoptosis, in part, due to enhanced aerobic glycolysis and biomass production, known as the Warburg effect. We previously demonstrated that combination simvastatin (SIM) and metformin (MET) ameliorates critical Warburg effect-related metabolic aberrations of C4-2B cells, synergistically and significantly decreases CRPC cell viability and metastatic properties, with minimal effect on normal prostate epithelial cells, and inhibits primary prostate tumor growth, metastasis, and biochemical failure in an orthotopic model of metastatic CRPC, more effectively than docetaxel chemotherapy. Several modes of cell death activated by individual treatment of SIM or MET have been reported; however, the cell death process induced by combination SIM and MET treatment in metastatic CRPC cells remains unknown. This must be determined prior to advancing combination SIM and MET to clinical trial for metastatic CRPC. Treatment of C4-2B cells with combination 4 *μ*M SIM and 2 mM MET (SIM+MET) led to significant G1-phase cell cycle arrest and decrease in the percentage of DNA-replicating cells in the S-phase by 24 h; arrest was sustained throughout the 96-h treatment. SIM+MET treatment led to enhanced autophagic flux in C4-2B cells by 72–96 h, ascertained by increased LC3B-II (further enhanced with lysosomal inhibitor chloroquine) and reduced Sequestosome-1 protein expression, significantly increased percentage of acidic vesicular organelle-positive cells, and increased autophagic structure accumulation assessed by transmission electron microscopy. Chloroquine, however, could not rescue CRPC cell viability, eliminating autophagic cell death; rather, autophagy was upregulated by C4-2B cells in attempt to withstand chemotherapy. Instead, SIM+MET treatment led to Ripk1- and Ripk3-dependent necrosis by 48–96 h, determined by propidium iodide-Annexin V flow cytometry, increase in Ripk1 and Ripk3 protein expression, necrosome formation, HMGB-1 extracellular release, and necrotic induction and viability rescue with necrostatin-1 and Ripk3-targeting siRNA. The necrosis-inducing capacity of SIM+MET may make these drugs a highly-effective treatment for apoptosis- and chemotherapy-resistant metastatic CRPC cells.

In the United States, ~30 000 men die each year from metastasis- and treatment-related complications of metastatic castration-resistant prostate cancer (CRPC).^1^ Depending on symptoms and prognosis, a variety of chemotherapies, immunotherapies, and radiotherapies are available;^2^ these treatment modalities modestly improve median progression-free and overall survival, but often with a toll on quality-of-life.^2, 3^ Another major issue is development of chemotherapeutic resistance.^3, 4^ More effective alternative therapies for metastatic CRPC are being sought.

One method of chemotherapeutic development involves taking advantage of aberrant cancer metabolism to target cancer cells while leaving normal cells unharmed. Aberrant cancer metabolism involves constitutive activity of the PI3K/Akt signaling pathway, inhibited adenosine monophosphate-activated protein kinase (AMPK) activity, and rampant aerobic glycolysis and glutamine utilization to significantly increase biomass synthesis necessary for accelerated cell growth, proliferation, and metastasis, known as the ‘Warburg effect'.^5, 6^ These metabolic alterations, including high p-Ser-473 Akt immunohistochemical staining and significantly elevated protein, fatty acid, and cholesterol concentrations (suggesting inhibited AMPK activity and increased macromolecule synthesis) were noted in primary prostate tumors of patients who progressed to biochemical failure or metastasis and in osseous metastases of prostate cancer.^7, 8, 9^ We also demonstrated constitutive activation of Akt, reduced AMPK activity, and significantly increased cholesterol synthesis in C4-2B *in vitro* models of metastatic CRPC.^10^

Exploiting metabolic aberrations present in CRPC cells for novel preclinical chemotherapeutic development, we devised a combination chemotherapy utilizing simvastatin (SIM) and metformin (MET).^10^ SIM is a potent inhibitor of 3-hydroxy-3-methylglutaryl-CoA reductase, the first rate-limiting enzyme of the mevalonate pathway,^11^ and MET is an indirect activator of AMPK, which acts by inhibiting the mitochondrial complex I and lowering the cellular ATP-to-AMP ratio.^12^ We previously demonstrated that 1 : 500 combination SIM+MET within pharmacological range significantly reduces Akt phosphorylation and increases AMPK activity, causing inhibition of downstream anabolic pathways, in C4-2B osseous metastatic CRPC cells.^10^ SIM+MET also synergistically inhibited CRPC cell viability and significantly abated metastatic properties *in vitro*, with minimal adverse effect on normal prostate epithelial cell viability, and significantly inhibited prostate primary tumor growth and prevented metastasis in an orthotopic model of metastatic CRPC.^10^ Suppression of Akt activity and reactivation of AMPK restrains aerobic glycolysis and macromolecule synthesis in cancer cells, inhibits proliferation and metastatic capability, and results in cell death.^5, 10^

SIM+MET treatment reduces C4-2B cell viability >90% by 96 h.^10^ SIM treatment was shown to induce cancer cell death by apoptosis, necrosis, or autophagy,^13, 14, 15^ and MET treatment leads to apoptosis or autophagy in prostate and other cancers.^16, 17^ The mode of cell death activated by combination SIM+MET in metastatic CRPC cells has not been elucidated, but is important to define prior to pursuing potential clinical application. Therefore, the purpose of this study was to determine the cell death process induced in C4-2B cells by SIM+MET treatment.

## Results

### SIM+MET treatment induces G1-phase cell cycle arrest in CRPC cells

**Figure 1:**
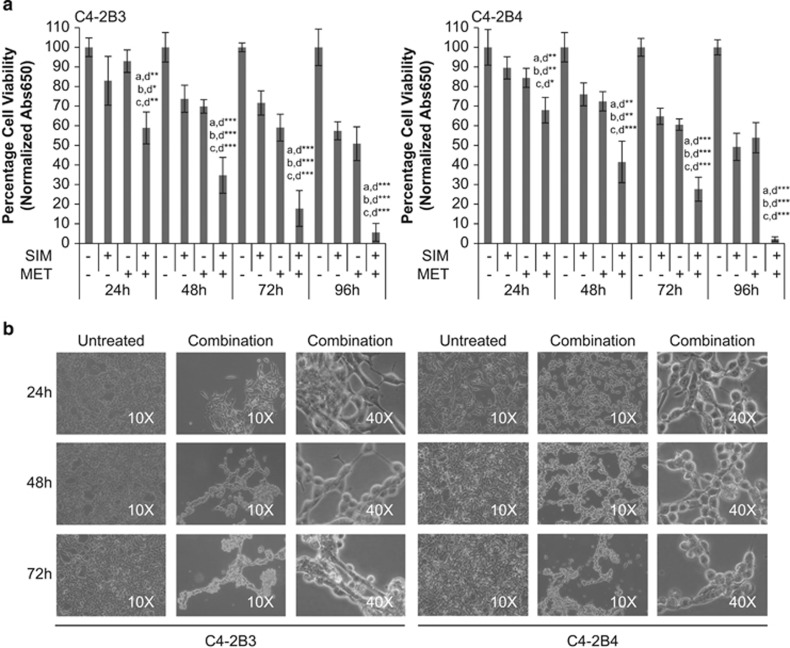
Combination simvastatin and metformin treatment significantly inhibits C4-2B metastatic CRPC cell viability. (**a**) Percentage cell viability (mean±S.D.) by the methylene blue assay in C4-2B3 and C4-2B4 cells following treatment with 4 *μ*M simvastatin (SIM) and/or 2 mM metformin (MET) for 24−96 h, *n*=3 per treatment group. *P<0.05, **P<0.01, ***P<0.001 determined by ANOVA followed by the Tukey multiple comparison procedure where a=untreated, b=SIM, c=MET, and d=SIM+MET in comparisons. (**b**) Light microscope images at × 10 and × 40 magnification of untreated and combination 4 *μ*M SIM and 2 mM MET-treated C4-2B3 and C4-2B4 cells at 24−72 h (96 h not shown as only 2−6% of SIM+MET-treated C4-2B cells remain viable at that time point)

**Figure 2:**
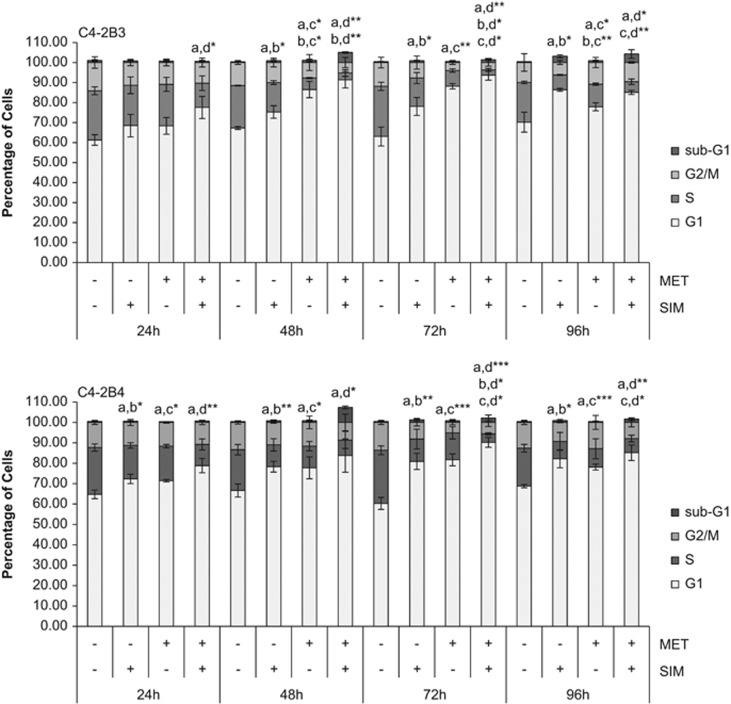
Combination simvastatin and metformin treatment induces significant, sustained G1-phase cell cycle arrest in C4-2B metastatic CRPC cells. C4-2B3 and C4-2B4 cells were treated with 4 *μ*M simvastatin (SIM) and 2 mM metformin (MET) for 24−96 h followed by staining with propidium iodide and analysis by flow cytometry. Percentage of cells (mean±S.D.) in G1, S, and G2/M phases of the cell cycle and sub-G1 represented in bar graphs, *n*=3 per treatment group. Significance of G1-phase cell cycle arrest, where *P<0.05, **P<0.01, ***P<0.001, determined by ANOVA followed by the Tukey multiple comparison procedure where a=untreated, b=SIM, c=MET, and d=SIM+MET in comparisons

SIM+MET treatment significantly inhibits C4-2B3/B4 cell viability, with 94–98% of cells nonviable by 96 h (Figures 1a and b). We previously demonstrated that SIM+MET significantly decreases Akt phosphorylation and activation and significantly increases AMPK*α* activity, starving C4-2B cells of macromolecules required for growth, proliferation, metastasis, and survival.^10, 18^ Inhibition of the glycolytic pathway and biomass synthesis often leads to cell cycle arrest.^19^ Individually, statins and MET induce cell cycle arrest in prostate cancer cells.^16, 17, 20^ Therefore, we first investigated whether SIM+MET treatment causes cell cycle arrest in C4-2B cells by propidium iodide (PI) DNA staining and flow cytometric analysis. Compared with untreated and SIM or MET individually treated C4-2B cells, SIM+MET treatment led to significant G1-phase arrest and decrease in percentage of DNA-replicating cells in the S-phase by 24 h treatment, and arrest was sustained throughout the 96-h treatment (Figure 2). Therefore, SIM+MET teatment led to an earlier, more pronounced, sustained, and significant G1-phase cell cycle arrest compared with SIM or MET treatment alone; this is sensible, as nutrient restriction generally leads to arrest at the G1-phase checkpoint.

### SIM+MET treatment does not induce apoptosis in CRPC cells

Sustained cell cycle arrest leads to cell death. Apoptosis is the dominant cell death process induced by SIM in prostate cancer cells;^13, 21^ MET also causes cell death by apoptosis in prostate cancer cells.^16^ With no prior reports regarding mechanism of cell death of combination SIM+MET in any cancer cell, we first investigated whether cell death is through apoptosis.

**Figure 3:**
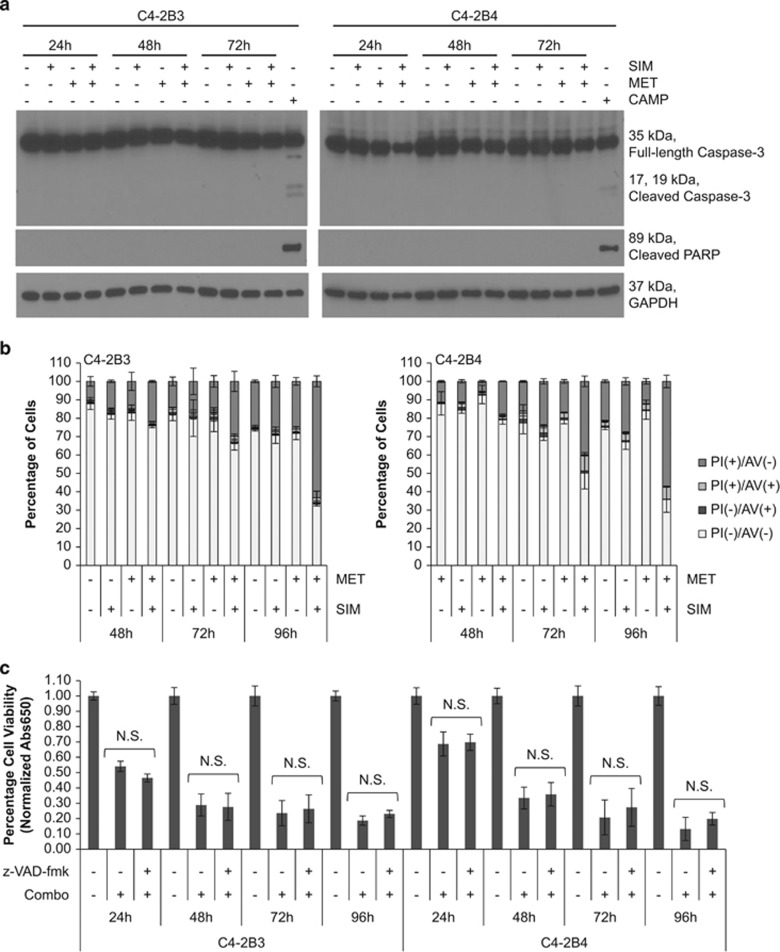
Combination simvastatin and metformin treatment does not induce apoptosis in C4-2B metastatic CRPC cells. (**a**) Western blot analysis of total and cleaved caspase-3 and cleaved PARP protein expression from total cell lysates of C4-2B3 and C4-2B4 cells following treatment with 4 *μ*M simvastatin (SIM) and/or 2 mM metformin (MET) for 24−72 h. Total cell lysates of C4-2B3 and C4-2B4 cells treated with 2 *μ*M (S)-(+)-camptothecin (CAMP) for 48 h used as apoptosis positive control. GAPDH used as loading control. (**b**) C4-2B3 and C4-2B4 cells treated with 4 *μ*M SIM and/or 2 mM MET for 48−96 h followed by staining with FITC-conjugated Annexin V (AV) and propidium iodide (PI) and analyzed by flow cytometry. Percentage of PI(−)AV(−), PI(−)AV(+), PI(+)AV(−), and PI(+)AV(+) cells demonstrated in bar graphs of mean±S.D. from triplicate samples. Representative density plots of controls and samples shown in Supplementary Figure S1. (**c**) Percentage cell viability (mean±S.D.) by methylene blue assay of C4-2B3 and C4-2B4 cells following treatment with combination 4 *μ*M SIM and 2 mM MET±10 *μ*M z-VAD-fmk pan-caspase inhibitor for 24−96 h, *n*=3 per treatment group. NS denotes not significant difference in cell viability between the treatment groups of SIM+MET in presence or absence of z-VAD-fmk as determined by the two-tailed Student's *t*-test

Apoptosis may be caused by toxins or nutrient deprivation and is characterized by cellular blebbing, cytoplasmic and nuclear shrinkage, chromatin condensation, and DNA fragmentation.^22, 23^ The spike (5.0–7.3%) of sub-G1 cells noted at 48-h SIM+MET treatment (Figure 2), indicative of DNA fragmentation, suggests some C4-2B cells may be undergoing apoptosis at this time point. Therefore, we evaluated the potential of SIM+MET to induce cell death by apoptosis using methods to capture potential activation of mitochondrial (intrinsic) and/or death receptor signaling (extrinsic) apoptotic pathways. Caspase-3 and downstream poly-(ADP-ribose) polymerase (PARP) are cleaved and activated by both pathways of apoptosis.^24^ In distinct contrast to apoptosis-inducing positive control (S)-(+)-camptothecin treatment, neither caspase-3 nor PARP cleavage was noted following SIM+MET treatment for 24–72 h (Figure 3a).

One of the earliest features of apoptosis involves translocation of phosphatidylserine from the internal to external bilayer of the cell membrane; Annexin V (AV) demonstrates high specificity and affinity for phosphatidylserine.^25^ Necrotic cells can also bind AV due to damaged plasma membranes; therefore, PI is used to distinguish between apoptotic and necrotic cells, as it can only enter unfixed cells across a damaged plasma membrane.^26^ In a PI/AV assay, PI(−)/AV(+) cells are apoptotic, PI(+)/AV(+) cells are either post-apoptotic secondary necrotic or primary necrotic, and PI(+)/AV(−) cells are necrotic; AV-positivity precedes PI-positivity in apoptotic cells, but coincides in necrotic cells (Supplementary Figures S1a and b).^27^ Following treatment with SIM+MET for 48–96 h, the percentage of viable PI(−)/AV(−) cells decreases significantly compared with untreated or individually treated cells; however, the percentage of PI(−)/AV(+) cells remains low (<4%), indicating C4-2B cells were not undergoing apoptosis (Figure 3b and Supplementary Figure S1c). Rather, AV- and PI-positivity of C4-2B cells occur simultaneously, with majority of cells becoming significantly PI(+)/AV(−), compared with untreated or individually treated cells. SIM+MET-treated C4-2B cells (21–24%) are PI(+)/AV(−) and PI(+)/AV(+) after 48 h, progressing to 64–66% after the 96-h treatment. Therefore, SIM+MET treatment does not induce apoptosis, but instead necrosis, markedly, by 72–96-h treatment.

As final confirmation that apoptosis is not the mode by which SIM+MET induces cell death in C4-2B cells, cells were treated with SIM+MET in presence and absence of 10 *μ*M z-VAD-fmk pan-caspase inhibitor for 24–96 h and viability assessed for rescue, which would indicate a caspase-dependent apoptotic cell death mechanism. The concentration 10 *μ*M z-VAD-fmk was chosen because it previously suppressed apoptosis in C4-2B cells without itself affecting viability^28^ and prevented caspase-3 and PARP cleavage in C4-2B cells treated with (S)-(+)-camptothecin (Supplementary Figure S2). Shown in Figure 3c, apoptosis suppression with z-VAD-fmk provided no cell viability rescue. Despite a temporary spike in hypoploid cells at 48 h of SIM+MET treatment (which could be due to other oncosis), our results do not support apoptosis as a cytotoxic pathway induced by SIM+MET treatment in C4-2B cells.

### CRPC cells upregulate autophagy in an attempt to withstand SIM+MET chemotherapy

Macroautophagy (hereafter referred to as autophagy) is a lysosomal-dependent process of self-digestion of cytoplasmic content regulated by AMPK that can have a role in mediating either cell survival or death in response to nutrient starvation and stress.^29^ Both SIM and MET can induce autophagy in prostate and other cancers.^15, 17^ We previously demonstrated that SIM+MET inhibit Akt phosphorylation and activity and reactivate AMPK activity in C4-2B cells;^10^ inhibition of Akt and activation of AMPK in cancer cells often leads to autophagy.^30^ Therefore, we next investigated autophagy as a potential mechanism by which SIM+MET induces cell death in C4-2B metastatic CRPC cells.

**Figure 4:**
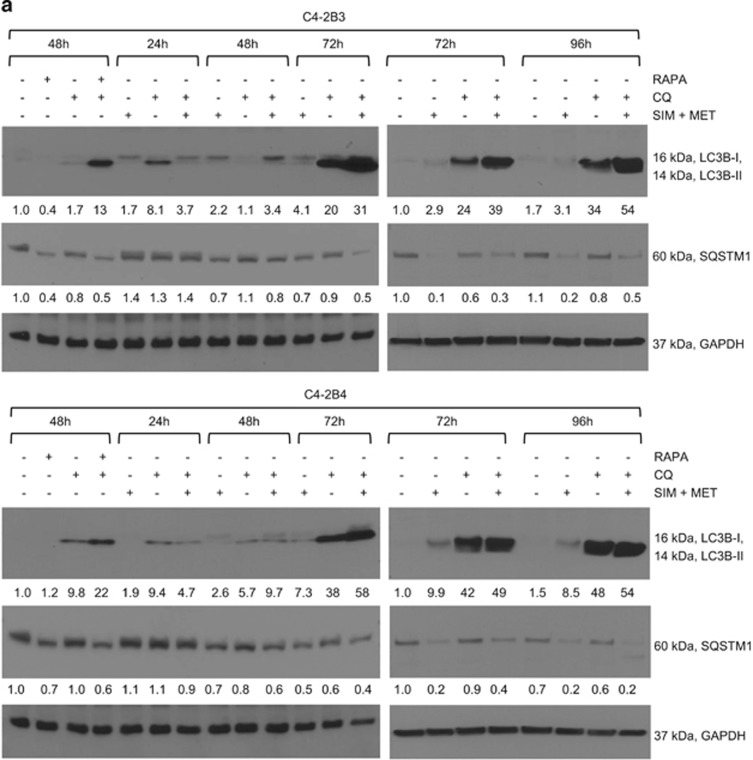

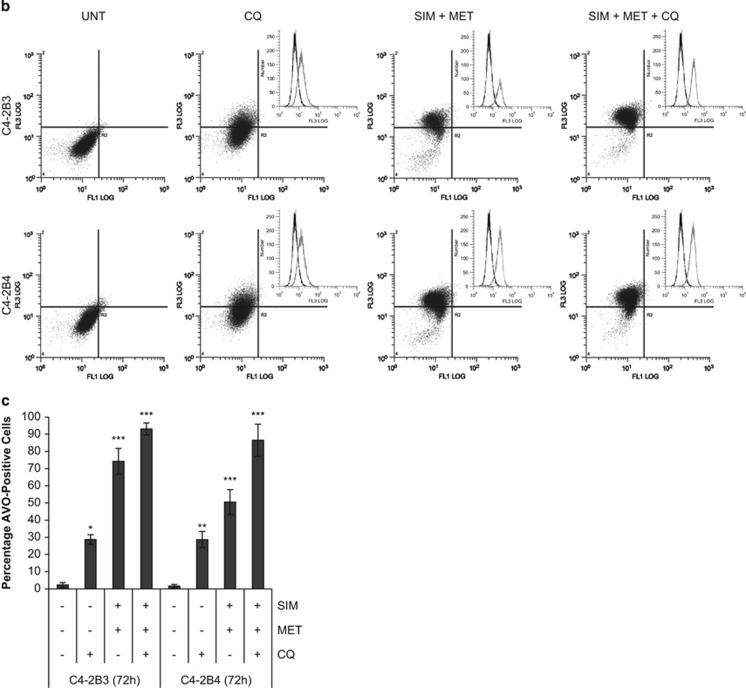
Combination simvastatin and metformin treatment increases autophagic flux in C4-2B metastatic CRPC cells. (**a**) Western blot analysis of LC3B-I and -II and sequestosome 1 (SQSTM1) protein expression in total cell lysates of C4-2B3 and C4-2B4 cells following treatment with combination 4 *μ*M simvastatin (SIM) and 2 mM metformin (MET) and/or 10 *μ*M chloroquine (CQ) lysosomal inhibitor for 24−96 h. Total cell lysates of C4-2B3 and C4-2B4 cells treated with 500nM rapamycin (RAPA) and/or 10 *μ*M CQ used as autophagic flux positive controls. GAPDH used as loading control. (**b**) Representative density plots of acidic vesicular organelles (AVOs) by acridine orange staining using flow cytometry in C4-2B3 and C4-2B4 cells treated with combination 4 *μ*M SIM and 2 mM MET±10 *μ*M CQ for 72 h. In acridine orange-stained cells, the nucleus fluoresces green (FL1, 525 nm emission, x-axis), whereas acidic compartments fluoresce red (FL3, 620 nm emission, y-axis). The intensity of red fluorescence is proportional to the degree of acidity and to the volume of AVOs, including autophagic vacuoles. Insets show increase in red fluorescence (FL3, x-axis) with treatment compared with untreated control. (**c**) Quantification (mean±S.D.) of percentages of cells from each treatment group from **b** with a significant proportion of AVOs, *n*=3 separate experiments. *P<0.05, **P<0.01, ***P<0.001 determined by a two-tailed Student's *t-*test compared with untreated control. Representative density plots and quantification of positive controls shown in Supplementary Figure S4

During autophagy, a phagophore engulfs a portion of cytoplasm containing organelles and proteins and encloses, forming an autophagosome, which then joins with a lysosome to form an autolysosome where contents are digested into reusable macromolecules.^31, 32^ Microtubule-associated protein 1 light chain 3 (LC3) is utilized as a marker of autophagic activity.^32, 33, 34^ There are three human isoforms of LC3, LC3A–C, which undergo post-translational modification during autophagy; LC3B being the homolog most commonly used to assess autophagy.^33, 34^ Following an autophagic trigger, cytosolic LC3B-I is conjugated with phosphatidylethanolamine (PE) to form LC3B-II, which is recruited to and detected on autophagosomal membranes.^32, 33, 34^ The quantity of LC3B-II is not static; during autophagosome formation, outer membrane LC3B-II is cleaved from PE by Atg4 to replenish available cytoplasmic LC3B-I and inner membrane LC3B-II degraded along with cargo within the autolysosome.^33^ LC3B-II protein expression is positively correlated with number of autophagosomes, and increase in LC3B-II is an indicator of autophagosome formation (autophagic flux).^33, 34^ However, increase in LC3B-II protein can also indicate inhibition of autolysosomal function.^33, 34^ To determine whether LC3B-II expression increases due to autophagic flux or autolysosomal function inhibition, a lysosomal inhibitor, such as chloroquine (CQ), is used.^34^ CQ is a lysosomotropic agent that becomes trapped within the lysosome due to protonation, and raises lysosomal pH, which inhibits lysosomal enzymes, prevents fusion of autophagosome and lysosome, and prevents lysosomal protein degradation. Therefore, an equivalent quantity of LC3B-II protein following SIM+MET treatment in presence and absence of CQ demonstrates inhibition of autolysosomal function, whereas enhanced LC3B-II accumulation in presence of CQ demonstrates that SIM+MET treatment induces autophagic flux. Shown in Figure 4a, LC3B-II accumulation is observed at 72–96-h SIM+MET treatment, but is only lightly visible due to LC3B-II recycling and degradation within the autolysosome. Yet, LC3B-II accumulation is greatly enhanced with SIM+MET treatment in presence of CQ, indicating that SIM+MET treatment induces autophagic flux, in a similar manner as positive control rapamycin-induced autophagy. The dosage 10 *μ*M CQ was chosen for ability to effectively inhibit autolysosome function without itself significantly affecting C4-2B cell viability (Supplementary Figures S3a and b). Another protein monitored to detect autophagic flux is sequestosome 1 (SQSTM1/p62), a scaffold protein that binds both ubiquitin and LC3B-II, and is integral in transport of ubiquitin-tagged protein aggregates to the autophagosome.^34, 35^ SQSTM1 is degraded along with cytoplasmic material within the autolysosome, leading to decreased SQSTM1 protein levels during autophagy.^34, 35^ As seen in Figure 4a, SQSTM1 protein markedly decreases following SIM+MET treatment for 72–96 h, and is partially rescued in presence of lysosomal inhibitor CQ, providing additional evidence of autophagic flux induction in response to SIM+MET treatment.

**Figure 5:**
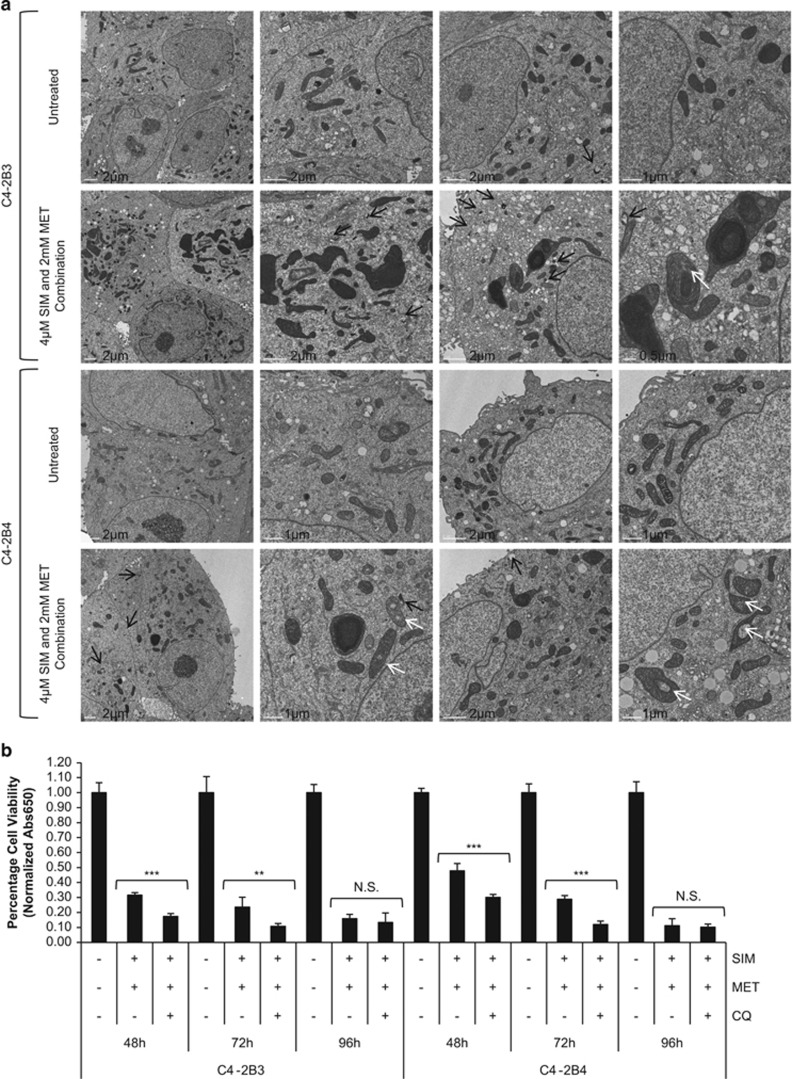
C4-2B metastatic CRPC cells upregulate autophagy as a protective mechanism against combination simvastatin and metformin treatment. (**a**) Transmission electron microscopy pictures of untreated and combination 4 *μ*M simvastatin (SIM) and 2 mM metformin (MET)-treated C4-2B3 and C4-2B4 cells at 72 h. Black arrows show double-membrane degradative (i.e., lysosome associated with vesicle) and lucent autophagic vesicles. White arrows show potentially mitoptotic mitochondria. Scale bar noted on images. (**b**) Percentage cell viability (mean±S.D.) by methylene blue in C4-2B3 and C4-2B4 cells following treatment with combination 4 *μ*M SIM and 2 mM MET±10 *μ*M chloroquine (CQ) lysosomal inhibitor for 48−96 h, *n*=3 per group. **P<0.01, ***P<0.001, and NS=no significant difference between treatment groups of SIM+MET in presence or absence of CQ, as determined by the two-tailed Student's *t-*test

An increase in the number of acidic vesicular organelles (AVOs), or intracellular acidic compartments such as autolysosomes and lysosomes, is also characteristic of autophagy.^36^ Acridine orange (AO) is an acidotropic dye that accumulates in AVOs and fluoresces bright red when protonated at low pH; the intensity of red fluorescence is proportional to the volume of AVOs and is quantified by flow cytometry (normalized to green fluorescence of AO-stained DNA/RNA).^36^ Autophagic induction following SIM+MET treatment for 72 h was confirmed by significantly increased percentage of AVO-positive C4-2B cells, which was further enhanced in presence of CQ, again indicating enhanced autophagic flux in response to SIM+MET treatment (Figures 4b and c) (percentage of AVO-positive cells in rapamycin±CQ-treated C4-2B3/B4 positive controls quantified in Supplementary Figure S4). In addition, transmission electron microscopy (TEM) revealed an accumulation of double-membrane degradative (lysosomes associated with vesicle, black arrows) and autophagic vesicles in C4-2B3/B4 cells following SIM+MET treatment for 72 h (Figure 5a).

Autophagy can be a mechanism of cell death; however, cancer cells also upregulate autophagy in an effort to withstand nutrient deprivation and potentially cytotoxic insults of drug treatment as a mechanism of chemoresistance.^37^ To determine whether the increase in autophagic flux observed in C4-2B cells following SIM+MET treatment for 72–96 h is an attempt to withstand chemotherapeutic insult or is part of the mechanism by which SIM+MET treatment induces C4-2B cell death, we sought to investigate whether autophagic inhibitor CQ could rescue C4-2B cell viability inhibition induced by SIM+MET treatment. Should CQ rescue viability, then autophagy is part of the cell death process induced by SIM+MET (i.e., autophagy-mediated necrotic cell death^38^), whereas lack of rescue or further reduction in C4-2B viability upon addition of CQ implies autophagy upregulated in C4-2B cells in attempt at cellular defense against SIM+MET chemotherapy. Following treatment with SIM+MET in presence of CQ, C4-2B cell viability was significantly further reduced at 48–72 h and unrescued at 96 h compared with viability in absence of CQ (Figure 5b); therefore, autophagy is upregulated by C4-2B cells following SIM+MET treatment as a mechanism of chemoresistance, but is not part of the cell death process.

### SIM+MET treatment induces necrotic cell death in CRPC cells

**Figure 6:**
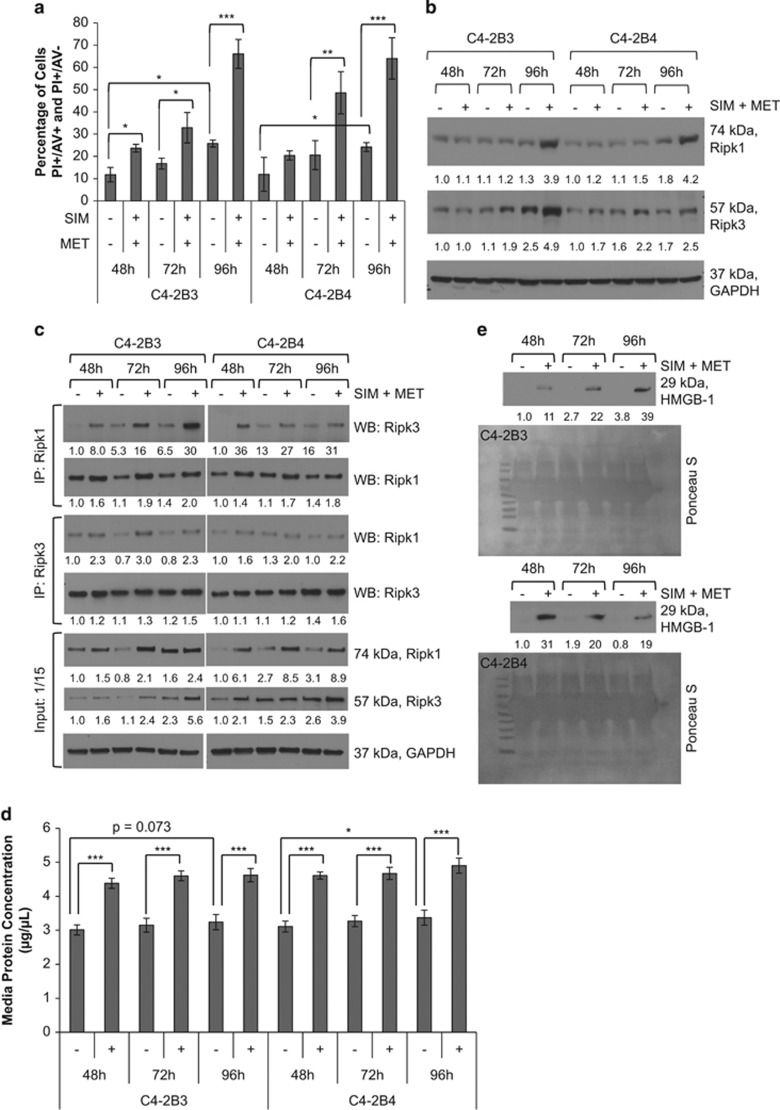
Combination simvastatin and metformin treatment induces necrotic cell death in C4-2B metastatic CRPC cells. (**a**) C4-2B3 and C4-2B4 cells treated with 4 *μ*M simvastatin (SIM) and 2 mM metformin (MET) for 48−96 h followed by staining with FITC-conjugated Annexin V (AV) and propidium iodide (PI) and analyzed by flow cytometry. Percentage of cells (mean±S.D.) staining PI+ (PI(+)AV(+) and PI(+)AV(−)) depicted in bar graphs, *n*=3 separate cytometric experiments. *P<0.05, **P<0.01, ***P<0.001 determined by ANOVA followed by the Tukey multiple comparison procedure. (**b**) Western blot analysis of Ripk1 and Ripk3 protein expression in total cell lysates of C4-2B3 and C4-2B4 cells following treatment with combination 4 *μ*M SIM and 2 mM MET for 48−96 h. GAPDH used as loading control. (**c**) Treatment of C4-2B3 and C4-2B4 cells with combination 4 *μ*M SIM and 2 mM MET for 48−96 h induces association of Ripk1 and Ripk3 in a time-dependent manner. Immunoprecipitation of 600 *μ*g protein from total cell lysates was conducted with Ripk1 and Ripk3 antibodies followed by western blot with Ripk1, Ripk3, and GAPDH antibodies. No GAPDH detected in immunoprecipitates (not shown). Forty *μ*g protein from total cell lysates were also immunoblotted as a control (input). Protein expression was quantified by densitometry normalized to GAPDH loading control (mean from two separate experiments). (**d**) Protein concentration (mean±S.D.) of conditioned media from C4-2B3 and C4-2B4 cells following treatment with combination 4 *μ*M SIM and 2 mM MET for 48−96 h, *n*=3 separate experiments. *P<0.05, ***P<0.001 determined by ANOVA followed by the Tukey multiple comparison procedure. (**e**) Western blot analysis of HMGB-1 protein in 40 *μ*l conditioned media from C4-2B3 and C4-2B4 cells following treatment with combination 4 *μ*M SIM and 2 mM MET for 48−96 h. HMGB-1 protein expression quantified by densitometry (mean from two separate experiments). Ponceau S stain used to demonstrate equal loading

We demonstrated through PI/AV assessment (Figures 3b and 6a,Supplementary Figure S1c) that SIM+MET treatment led to significant increase in PI(+) C4-2B cells by 48−96 h; in fact, at the 96-h combination treatment, 61−64% of C4-2B cells were either PI(+)/AV(−) or PI(+)/AV(+), strongly implicating necrosis as the primary cell death process associated with SIM+MET treatment in C4-2B cells. SIM treatment was shown to induce necrotic cell death in cancer cells, including prostate cancer, albeit at supraphysiological concentrations of inactivated SIM.^14, 39^ Necrosis is cell death in response to external factors, characterized by cell clumping and swelling (C4-2B cells are clumped and swollen at 48−72-h SIM+MET treatment in Figure 1b), protein denaturation, nuclear membrane, organelle, and plasma membrane disruption, and release of cellular material into extracellular space.^29^ Necrosis was recently discovered to be a regulated mode of cell death of which the central mediators are serine–threonine kinases receptor-interacting protein 1 (Ripk1) and Ripk3.^29, 40^ Upon the necrosis-stimulating trigger, Ripk1 and Ripk3 bind one another, are phosphorylated and activated, form a complex with caspase-8, FADD, and other proteins, called a necrosome, and necrosis is induced.^29, 41, 42^

To confirm SIM+MET induces necrotic cell death in C4-2B cells, we first investigated the effect of SIM+MET treatment on protein expression of Ripk1 and Ripk3, as expression of these kinases increases as cells undergo necrosis.^43^ Shown in Figure 6b, both Ripk1 and Ripk3 protein expression increased in C4-2B cells following combination treatment for 72−96 h. To confirm Ripk1–Ripk3 interaction and necrosome formation, Ripk1 and Ripk3 were immunoprecipitated from C4-2B3/B4 whole-cell lysates following SIM+MET treatment for 48−96 h; increased co-precipitation of Ripk1 and Ripk3 was found in both C4-2B strains following combination treatment (Figure 6c). Cells undergoing necrosis release large quantities of protein into the extracellular space.^27, 44^ A 42−48% increase in protein concentration of conditioned media from C4-2B cells treated with SIM+MET for 48−96 h was observed compared with untreated cells (Figure 6d), indicative of large-scale protein release possibly related to necrotic induction. Immunoblotting of conditioned media also revealed readily detected high-mobility group box-1 (HMGB-1) protein following SIM+MET treatment for 48−96 h (Figure 6e); HMGB-1 is nuclear-localized in viable cells, but released extracellularly during necrotic rupture.^27^ Of note, a basal level of necrotic characteristics exists in C4-2B cells; 11.8−25.8% of untreated cells are PI(+), significantly increasing with time (Figure 6a), and Ripk1 and Ripk3 protein expression (Figure 6b) and necrosome formation (Figure 6c) also increases throughout the timecourse in untreated cells.

### SIM+MET-induced necrosis in CRPC cells is Ripk1- and Ripk3 dependent

**Figure 7:**
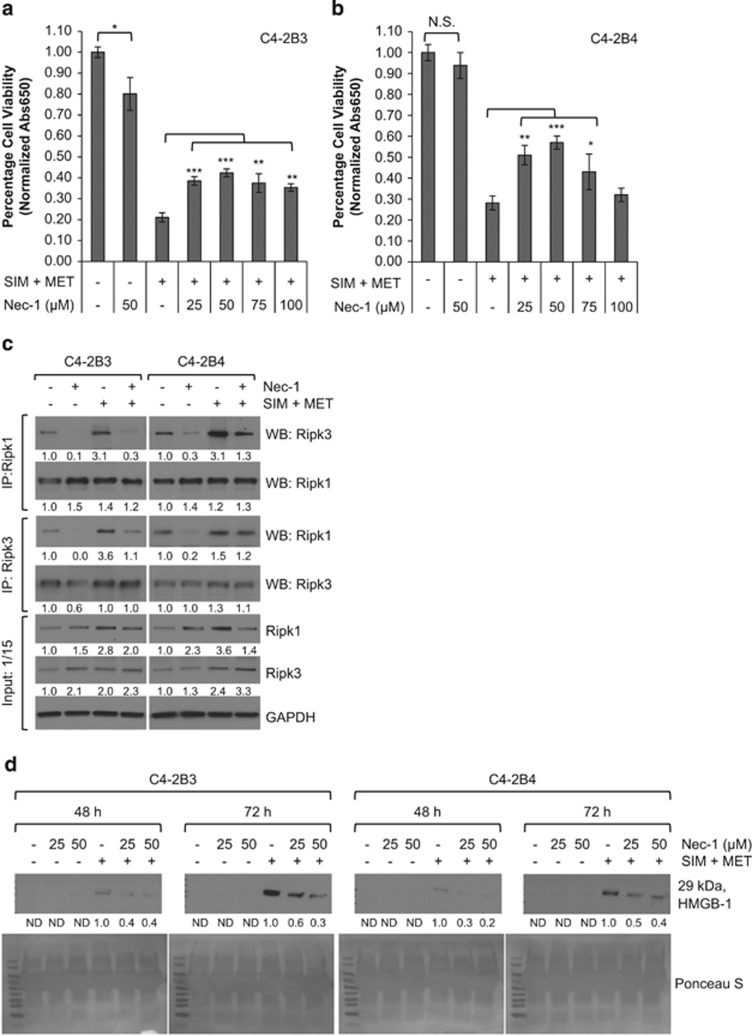
SIM+MET-induced necrosis in C4-2B metastatic CRPC cells is in part Ripk1 dependent. (**a**,**b**) Percentage cell viability (mean±S.D.) by the methylene blue assay in C4-2B3 and C4-2B4 cells following treatment with 50 *μ*M Ripk1 inhibitor necrostatin-1 (Nec-1) or combination 4 *μ*M simvastatin (SIM) and 2 mM metformin (MET)±25−100 *μ*M Nec-1 for 72 h, *n*=3 separate experiments. *P<0.05, **P<0.01, ***P<0.001, NS=not significant, as determined by ANOVA followed by the Tukey multiple comparison procedure. (**c**) Treatment of C4-2B3 and C4-2B4 cells with 50 *μ*M Nec-1 for 72 h reduces Ripk1–Ripk3 association. Immunoprecipitation of 600 *μ*g protein from total cell lysates of C4-2B3 and C4-2B4 cells treated with 50 *μ*M Nec-1 and/or combination 4 *μ*M SIM and 2 mM MET was conducted with Ripk1 and Ripk3 antibodies followed by western blot with Ripk1, Ripk3, and GAPDH antibodies. No GAPDH detected in immunoprecipitates (not shown). Protein (40 *μ*g) from total cell lysates were also immunoblotted as a control (input). Protein expression was quantified by densitometry normalized to GAPDH loading control (mean from two separate experiments). (**d**) Western blot analysis of HMGB-1 protein in 40 *μ*l conditioned media from C4-2B3 and C4-2B4 cells following treatment with 25−50 *μ*M Nec-1 or combination 4 *μ*M SIM and 2 mM MET±25−50 *μ*M Nec-1 for 48−72 h. HMGB-1 protein expression quantified by densitometry (mean from two separate experiments). ND, none detected. Ponceau S stain used to demonstrate equal loading

**Figure 8:**
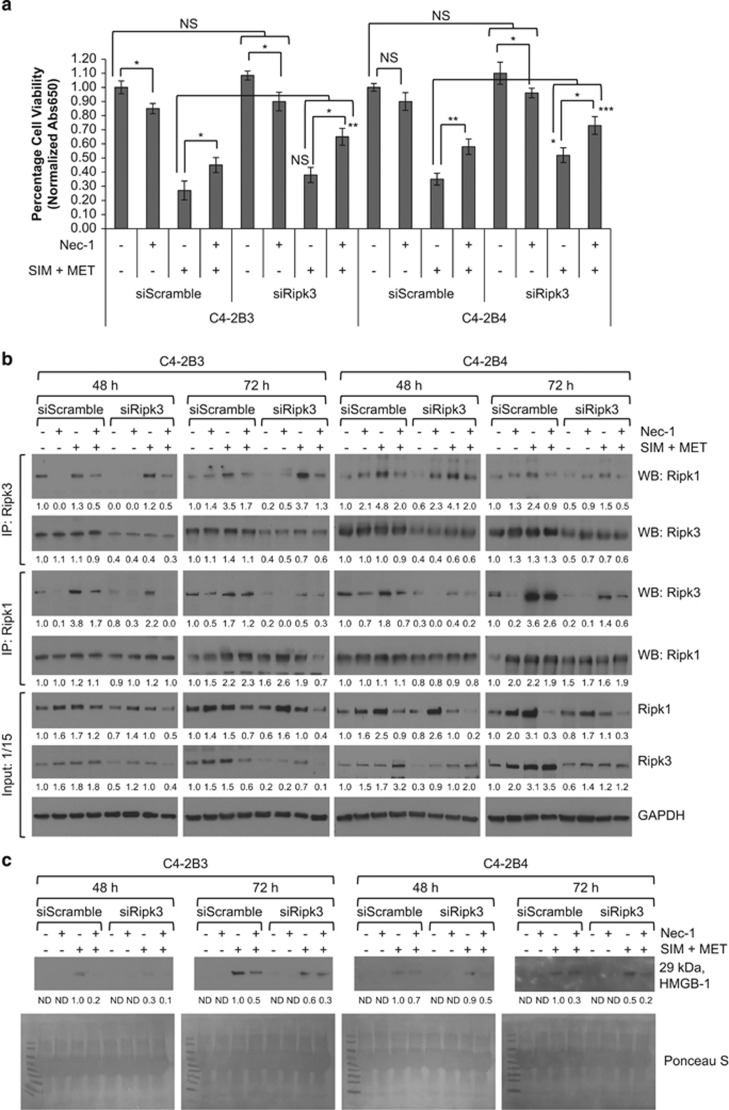
SIM+MET-induced necrosis in C4-2B metastatic CRPC cells is both Ripk1- and Ripk3 dependent. (**a**) Percentage cell viability (mean±S.D.) by the methylene blue assay in non-targeting (siScramble) and Ripk3-targeting (siRipk3) siRNA transfected C4-2B3 and C4-2B4 cells following treatment with 50 *μ*M Ripk1 inhibitor necrostatin-1 (Nec-1) or combination 4 *μ*M simvastatin (SIM) and 2 mM metformin (MET)±25−100 *μ*M Nec-1 for 72 h, *n*=3 separate experiments. *P<0.05, **P<0.01, ***P<0.001, NS=not significant, as determined by ANOVA followed by the Tukey multiple comparison procedure. (**b**) Treatment of C4-2B3 and C4-2B4 cells with 50 *μ*M Nec-1 for 72 h and siRNA knockdown of Ripk3 expression additively reduces Ripk1–Ripk3 association. Immunoprecipitation of 600 *μ*g protein from total cell lysates of non-targeting (siScramble) and Ripk3-targeting (siRipk3) siRNA-transfected C4-2B3 and C4-2B4 cells treated with 50 *μ*M Nec-1 and/or combination 4 *μ*M SIM and 2 mM MET was conducted with Ripk1 and Ripk3 antibodies followed by western blot with Ripk1, Ripk3, and GAPDH antibodies. No GAPDH was detected in immunoprecipitates (not shown). Forty *μ*g protein from total cell lysates were also immunoblotted as a control (input). Protein expression was quantified by densitometry normalized to GAPDH loading control (mean from two separate experiments). Ripk3 siRNA resulted in 62−64% knockdown in Ripk3 protein expression at 48 h and 48−56% knockdown at 72 h. (**c**) Western blot analysis of HMGB-1 protein in 40 *μ*l conditioned media from non-targeting (siScramble) and Ripk3-targeting (siRipk3) siRNA-transfected C4-2B3 and C4-2B4 cells following treatment with 50 *μ*M Nec-1 and/or combination 4 *μ*M SIM and 2 mM MET for 48−72 h. HMGB-1 protein expression quantified by densitometry (mean from two separate experiments). ND, none detected. Ponceau S stain used to demonstrate equal loading

Ripk1 and Ripk3 can be mutually or individually responsible for necrotic induction,^45, 46, 47^ so we next determined whether SIM+MET-induced necrosis was Ripk1- and/or Ripk3 dependent using necrostatin-1 (Nec-1), a Ripk1 activity inhibitor, and siRNA to knockdown Ripk3 expression (resulted in average 62−67% knockdown of Ripk3 protein expression at 48 h and 56−66% knockdown at 72 h). Nec-1, in a dose-dependent manner to 50 *μ*M, partially rescued C4-2B viability from the effect of SIM+MET treatment at 72 h (Figures 7a and b); however, efficacy of Nec-1 rescue was lost at subsequent higher dosages. In fact, treatment of C4-2B3 cells with 50 *μ*M Nec-1 significantly decreased viability by 20% (Figure 7a); Ripk1 activity inhibition may affect cell survival because of Ripk1 important role as a common link in apoptotic, necrotic, and survival signaling pathways.^48^ Nec-1 also effectively inhibited Ripk1–Ripk3 co-precipitation (Figure 7c) and HMGB-1 extracellular release into conditioned media in a dose-dependent manner (Figure 7d). In siScramble- or siRipk3-transfected C4-2B cells, Nec-1 and Ripk3 knockdown each partially rescued C4-2B viability by 18−23% and 11−17%, respectively (Figure 8a); yet, when Ripk3 protein expression and Ripk1 activity were simultaneously inhibited, it resulted in highly significant 36−42% rescue of C4-2B cell viability from the effect of SIM+MET treatment for 72 h. Simultaneous Ripk3 protein expression and Ripk1 activity inhibition also effectively inhibited Ripk1–Ripk3 co-precipitation (Figure 8b) and HMGB-1 release (Figure 8c) in a time-dependent manner, with a combined effect on the inhibition of SIM+MET-induced necrosis greater than either Ripk3 siRNA or Nec-1 alone, indicative of a Ripk1- and Ripk3-dependent necrotic cell death mechanism.

## Discussion

We determined that SIM+MET treatment in C4-2B metastatic CRPC cells induces sustained G1-phase cell cycle arrest (24−96 h) leading to Ripk1- and Ripk3-dependent necrotic cell death by 72−96 h. In addition, autophagy is upregulated in C4-2B cells by 72−96 h of SIM+MET treatment as a mode of chemoresistance. From a metabolic standpoint, the mode of cell death induced by SIM+MET in C4-2B cells is sensible.

SIM+MET treatment inhibits Akt phosphorylation and activity in C4-2B cells, which decreases inhibitory Ser-485/491 phosphorylation on AMPK*α*_1_/*α*_2_ and reactivates AMPK*α*.^10^ In addition, MET indirectly activates AMPK by mitochondrial complex I inhibition and decrease of cellular ATP,^12^ and SIM activates AMPK by depletion of cellular geranylgeranyl diphosphate.^30^ SIM+MET treatment and subsequent AMPK activation inhibits aerobic glycolysis and macromolecule synthesis,^6, 10, 49^ impeding CRPC growth, proliferation, and metastasis.^5, 10^ The lack of nutrients starves the C4-2B CRPC cell, and prevents progression beyond the G1-phase cell cycle checkpoint, leading to G1-phase cell cycle arrest.

After prolonged AMPK activation and starvation, autophagy, as a protective mechanism, and cell death are triggered.^50^ C4-2B cells were shown to be resistant to extrinsic apoptotic cell death (i.e., TNFR-family-induced apoptotic cell death) due to loss of tumor necrosis factor receptor-associated death domain (TRADD) mRNA and protein expression.^51^ Loss of TRADD expression may explain why C4-2B cells are resistant to docetaxel-induced cell death,^10^ as taxanes induce death receptor-mediated apoptotic cell death,^52^ and may also explain why we did not observe apoptotic induction in C4-2B cells following SIM+MET treatment. It has been demonstrated that if an apoptotic block exists downstream of death receptors, activation instead leads to necrosis,^48, 53^ since Ripk1, a scaffold protein associated with death receptors, links survival, apoptosis, and necrosis pathways.^48^ If death receptors are triggered and apoptosis blocked, Ripk1 and Ripk3 bind one another, form the necrosome, and induce necrotic cell death,^29, 41, 42, 48^ as was observed with SIM+MET treatment in C4-2B cells. Although the spike of hypoploid cells at the 48-h combination treatment is interesting, our results do not support apoptosis as a cytotoxic pathway induced by SIM+MET treatment in C4-2B cells. Sub-G1 cells could be caused by another oncosis, perhaps from the necrosis effect on surrounding cells, nuclear fragments, clusters of chromosomes, micronuclei, or nuclei with normal DNA content but altered chromatin structure and diminished accessibility of PI fluorochrome to DNA.^54, 55, 56^ Also interesting, C4-2B cells were necrotic even in an untreated state; 12% of C4-2B cells were PI(+) at 48 h significantly increasing to 24% at 96 h, and Ripk1 and Ripk3 expression and necrosome formation also increased with timecourse; this is not surprising, as necrotic cells are often observed in prostate tumors following castration and in metastases.^56^

Cells employ autophagy to maintain cellular metabolism under starvation conditions and to remove damaged organelles under stress conditions to improve survival.^52^ We demonstrated that C4-2B cells upregulate autophagy in an attempt to survive SIM+MET chemotherapy. This was not a failed attempt as, in presence of autophagic inhibitor CQ, C4-2B cell viability was significantly reduced. Upregulation of autophagy prevented C4-2B cells from dying more rapidly, but was eventually overcome (no viability difference between SIM+MET±CQ-treated cells by 96 h). If another drug is used in combination with SIM+MET to block autophagic flux without affecting increased AMPK activity, perhaps targeting ULK1 and ULK2 downstream of AMPK, chemotherapeutic applicability of SIM+MET may be further improved and lead to more pronounced CRPC cell death.

The mitochondrial changes noted in SIM+MET-treated C4-2B cells are interesting and require further investigation. SIM+MET treatment appears to prevent mitochondrial division, as indicated by fewer mitochondria, fewer dividing mitochondria in treated cells, and large mitochondria with what appear to be multiple bodies within the mitochondrial membrane (Figure 5a). SIM+MET treatment may be inhibiting mitochondrial complex I activity, coenzyme Q10 and ATP synthesis, and inducing oxidative stress-induced mitochondrial dysfunction;^12, 14, 39^ it is also unknown whether these mitochondrial changes are effects of SIM or MET treatment alone or, more likely, a combinatorial effect. Further investigation will also be needed to determine whether mitoptosis is occurring (Figure 5a, white arrows) or whether these areas are cytoplasmic inclusions. Low cellular levels of ATP favor cell death by necrosis, as was observed with SIM+MET treatment in C4-2B cells, whereas complete ATP depletion triggers mitoptosis, which is frequently observed in conjunction with necrosis.^57^ Mitochondrial dysfunction and mitoptosis can also lead to mitophagy; although not observed in the single-time-point TEM images of C4-2B3/B4 at 72-h SIM+MET treatment, autophagic flux upregulation observed at 72−96-h combination treatment may be in part due to mitophagy of abnormal mitochondria. The mechanism of how SIM+MET induces these mitochondrial alterations in CRPC cells will be investigated further.

Because normal cells do not possess the same metabolic aberrations as metastatic CRPC cells, SIM+MET treatment had only a minimal effect on normal prostate epithelial cell viability, ~5% decrease in viability at the top-end of the pharmacological range.^10^ In stark contrast, SIM+MET treatment significantly inhibits metastatic CRPC cell viability and metastatic properties, ameliorates CRPC metabolic aberrations, and induces cell cycle arrest and necrotic cell death in CRPC cells. Although C4-2B cells are resistant to cell death by extrinsic apoptosis, they are not resistant to necrotic cell death, making SIM+MET an excellent novel potential chemotherapeutic option for apoptosis- and chemotherapy-resistant metastatic CRPC cells.

## Materials and methods

### Cell culture and conditions

C4-2B3 and C4-2B4 cells were a generous gift from Dr. Robert A Sikes, University of Delaware, and were maintained in 10% FBS (Invitrogen Life Technologies, Grand Island, NY, USA) 1% penicillin–streptomycin (HyClone, Fisher Scientific, Pittsburgh, PA, USA) RPMI-1640 (HyClone, Fisher Scientific) in a 37-°C incubator with an atmosphere of 5% CO_2_.

### Reagents

SIM and MET (AK Scientific, Union City, CA, USA); RNase A (Fisher Scientific); z-VAD-fmk (BD Biosciences, San Diego, CA, USA); PI, (S)-(+)-camptothecin, rapamycin, CQ, and AO (Sigma-Aldrich, St. Louis, MO, USA); necrostatin-1 (R&D Systems, Richfield, MN, USA). The prodrug simvastatin was activated to simvastatin acid before *in vitro* use.^10^

### Methylene blue assay

Assay was performed as described previously.^10^ Briefly, cells were cultured in 24-well plates; following treatment, cells were washed with PBS, stained with 2 g/l methylene blue solution for 1 h, and excess stain removed with ddH_2_O. For semi-quantification, bound methylene blue was eluted with 0.1N HCl with shaking, and absorbance measured spectrophotometrically at *λ*=650 nm (FLUOstar Omega, BMG Labtech, Ortenburg, Germany).

### Microscope images

Following treatment for 24−72 h, images captured at × 10 and × 40 magnification using an Olympus CKX41 microscope and DP12 digital microscope camera (Olympus America, Melville, NY, USA).

### Cell cycle analysis by PI flow cytometry

C4-2B cells were serum-starved overnight to synchronize, treated with 4 *μ*M SIM and/or 2 mM MET for 24−96 h, trypsinized, washed twice with cold 1 × PBS, and 1 × 10^6^ cells fixed and permeablized with 90% cold methanol overnight at −20 °C. Cells were then incubated at 37 °C with 20 *μ*g/ml RNase A in 1 × PBS for 30 min, stained with 50 *μ*g/ml PI for 30 min, and analyzed using an Epics XL cytometer (Beckman Coulter, Miami, FL, USA), EXPO32 acquisition software (version 12, Verity Software House Inc., Topsham, ME, USA), and WinList analysis software (version 7, Verity Software House Inc).

### Western blot analysis

Total cell lysates of exponentially growing cells were prepared by homogenization using stainless steel beads (Next Advance, Averill Park, NY, USA) as described previously.^10^ Forty *μ*g of protein was denatured at 95 °C, resolved over 4–20% SDS-PAGE (Bio-Rad, Hercules, CA, USA), and transferred to a nitrocellulose membrane. Following Ponceau S visualization and blocking with 5% nonfat dry milk TBST, pH 7.4 (USB Molecular Biology Reagents, Affymetrix, Cleveland, OH, USA) for 1 h, the membrane was probed with primary antibody overnight at 4 °C (Supplementary Table S1), incubated with corresponding HRP-conjugated secondary antibody (Santa Cruz Biotechnology, Santa Cruz, CA, USA), and detected using Pierce ECL reagent (Fisher Scientific). Bands were visualized upon autoradiography film (Denville Scientific, Metuchen, NJ, USA) exposure, quantified using Image J software (NIH, Bethesda, MD, USA), and normalized to the loading control. Total cell lysates for autophagic analysis were prepared with 2% Triton-X 100 lysis buffer, in order to properly extract hydrophobic LC3B-II,^32^ and resolved over a 12% SDS-PAGE. For western blot analysis of media protein, 40 *μ*l conditioned media per sample was resolved and transferred as above, and blots were stained with Ponceau S to demonstrate equal loading and photographed.

### PI/AV flow cytometric analysis

Assay performed according to the manufacturer's instructions (Annexin V-FITC Apoptosis Detection Kit, Phoenix Flow Systems, San Diego, CA, USA). Briefly, C4-2B cells were serum-starved overnight, treated with 4 *μ*M SIM and/or 2 mM MET for 48−96 h, trypsinized, washed with 1 × PBS, and 1 × 10^6^ cells incubated and stained with AV and PI, and analyzed using an Epics XL cytometer (Beckman Coulter), EXPO32 acquisition software, and WinList analysis software (version 7, Verity Software House Inc.) alongside control samples.

### AO staining for AVOs detection by flow cytometry

Following indicated treatment, C4-2B3 and C4-2B4 cells were stained with 1 *μ*g/ml AO in complete media for 15 min, then washed with 1 × PBS, trypsinized, resuspended at 1 × 10^6^ cells per ml cold 1 × PBS, and analyzed by flow cytometry. For quantization of percentage of AVO-positive cells, red (620 BP, FL3 channel, where BP=band pass) and green (525 BP, FL1 channel) fluorescence emissions from 3 × 10^4^ cells from samples and control specimens illuminated with blue (488nm) excitation light was analyzed using an Epics XL cytometer (Beckman Coulter), EXPO32 acquisition software, and WinList analysis software (version 7, Verity Software House Inc.), according to published protocol.^36, 58^

### Transmission electron microscopic analysis

C4-2B3 and C4-2B4 cells (1 × 10^5^) were seeded onto 3.0 mg/ml Matrigel (BD Biosciences)-coated sterilized Aclar Embedding Film (Electron Microscopy Sciences, Hatfield, PA, USA) lining the wells of 35-mm culture plates, then treated with combination 4 *μ*M SIM and 2 mM MET or untreated for 72 h. Cells were then fixed and processed for TEM according to the published protocol^59^ and analyzed using a FEI T12 Tecnai Spirit electron microscope (FEI, Hillsboro, OR, USA).

### Immunoprecipitation

Ripk1 and Ripk3 were immunoprecipitated from 600 *μ*g pre-cleared whole-cell lysate as per the manufacturer's protocol using 1.0 *μ*g primary antibody and 20 *μ*l Protein G-Agarose (Roche Diagnostics, Indianapolis, IN, USA) and Protein A/G PLUS-Agarose (Santa Cruz Biotechnology) conjugate suspension, respectively. Immunoprecipitates were washed four times, reduced, boiled, and resolved by SDS-PAGE as described above. Immunoprecipitate blots were probed to confirm absence of GAPDH to ensure proper separation and pull down.

### Quantization of conditioned media protein

Protein concentration (*μ*g/*μ*l) assessed in 5 *μ*l conditioned media (performed in triplicate) using the Lowry protein assay kit (Bio-Rad).

### RNA interference of Ripk3 expression

Cells were transfected with 40 pmol either SMARTpool ON-TARGETplus Ripk3 siRNA or ON-TARGETplus non-targeting siRNA (Thermo Scientific Dharmacon) using X-tremeGENE 9 DNA Transfection Reagent (Roche Diagnostics) as per the manufacturer's protocol.

### Statistics

Quantitative values represented as mean±S.D. of at least three independent experiments. Significance was determined by a two-tailed, unpaired Student's *t-*test or ANOVA, followed by the Tukey multiple comparison procedure (SAS 9.3, version 9.3, SAS Institute Inc., Cary, NC, USA). Comparisons resulting in *P*<0.05 considered statistically significant and identified with an asterisk (*), *P*<0.01 identified with double asterisk (**), and *P*<0.001 identified with triple asterisk (***).

## Abbreviations

AMPK: adenosine monophosphate-activated protein kinase
AO: acridine orange
AV: Annexin V
AVOs: acidic vesicular organelles
CQ: chloroquine
CRPC: castration-resistant prostate cancer
GAPDH: glyceraldehyde-3-phosphate dehydrogenase
LC3: microtubule-associated protein 1 light chain 3
MET: metformin
Nec-1: necrostatin-1
PARP: poly (ADP-ribose) polymerase
PE: phosphatidylethanolamine
PI: propidium iodide
Ripk1 and Ripk3: receptor-interacting protein kinase 1 and 3
SIM: simvastatin
SQSTM1: sequestosome 1
TEM: transmission electron microscopy
TRADD: tumor necrosis factor receptor-associated death domain
