# Supplementary material for: Combination simvastatin and metformin induces G1-phase cell cycle arrest and Ripk1- and Ripk3-dependent necrosis in C4-2B osseous metastatic castration-resistant prostate cancer cells
Source: Cell Death Dis. 2014 Nov 20;5(11):e1536–. doi: 10.1038/cddis.2014.500 (PMC4260755; doi:10.1038/cddis.2014.500)
Supplement: Supplementary Information [file cddis2014500x1.pdf]

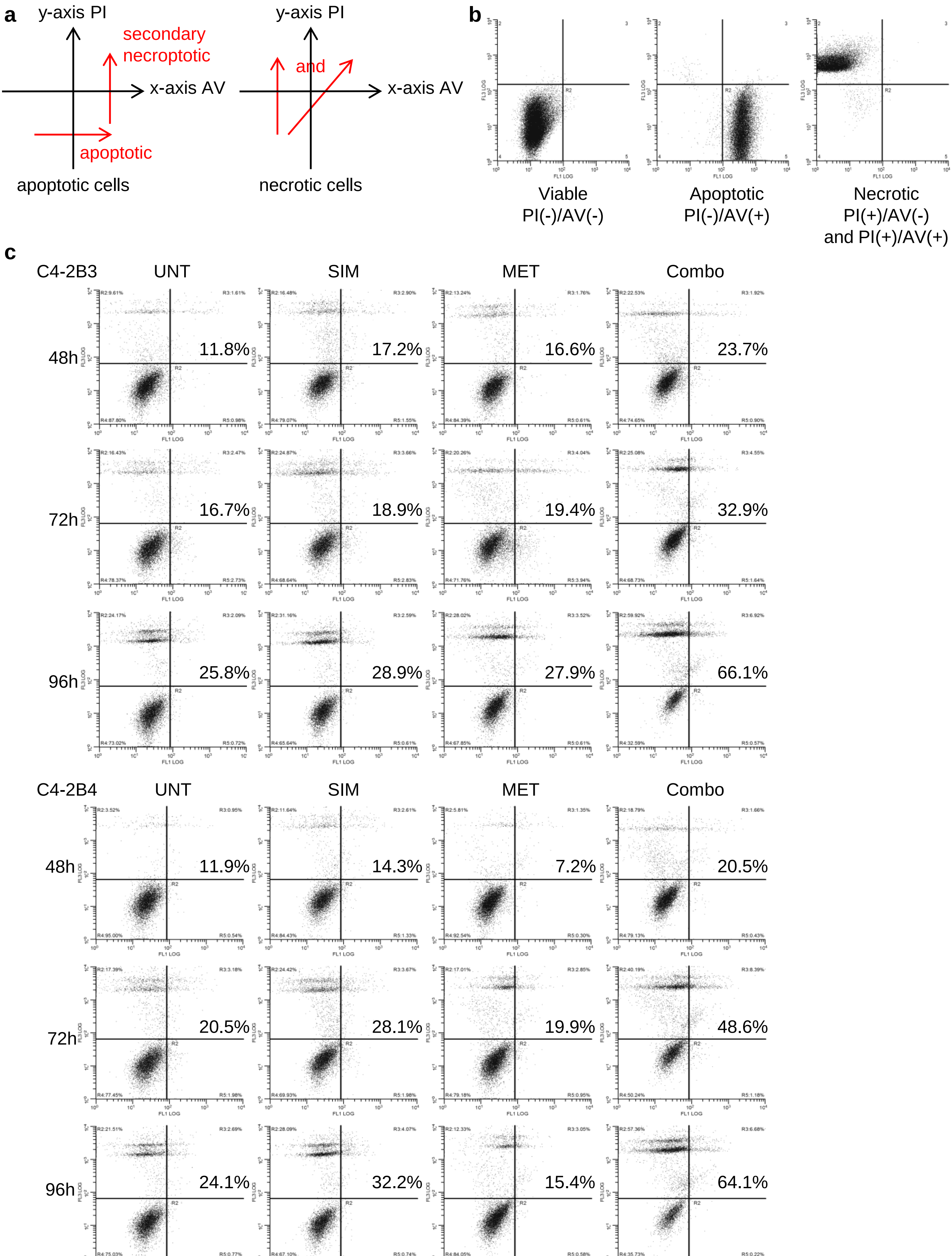

Supplementary Figure S1

Supplementary Figure S1. (previous page) Combination SIM and MET induces necrosis in C4-2B3 and C4-2B4 metastatic CRPC cells. (**a**) In apoptotic and necrotic cell death analysis by propidium iodide (PI) and FITC-conjugated Annexin V (AV) flow cytometry, apoptotic cells stain positive for AV prior to staining positive for PI; whereas, in necrotic cells, PI positivity and AV positivity coincide. (**b**) Representative PI/AV assay negative and positive controls. Viable cells have low PI and AV staining (lower-left quadrant), apoptotic cells have low PI and high AV staining (lower-right quadrant), and necrotic cells have high PI staining, and may or may not be AV positive (upper quadrants). (**c**) C4-2B3 and C4-2B4 cells treated with 4µM SIM and/or 2mM MET for 48-96h, followed by staining with AV and PI and analyzed by flow cytometry. Results are shown as density plots with AV x-axis and PI y-axis. One representative experiment of three is shown. Mean percentage of PI(+) cells stated on plot. UNT, untreated; SIM, 4µM simvastatin; MET, 2mM metformin; Combo, combination 4µM simvastatin and 2mM metformin.

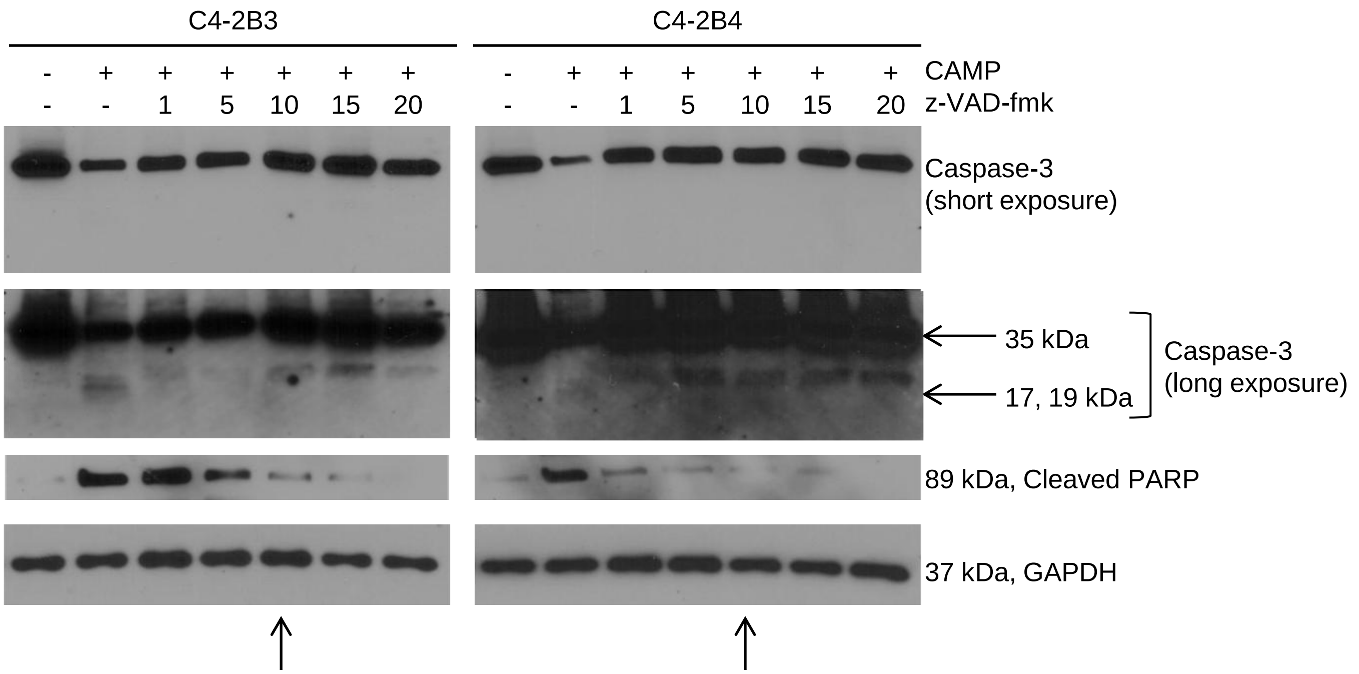

Supplementary Figure S2. 10µM z-VAD-fmk prevents caspase-3 and PARP cleavage induced by 2µM (S)-(+)-camptothecin treatment in C4-2B3 and C4-2B4 metastatic CRPC cells. Western blot analysis of total cell lysates from C4-2B3 and C4-2B4 cells following treatment with 2µM (S)-(+)-camptothecin (CAMP) ± 1-20 µM z-VAD-fmk pan-caspase inhibitor for 48h. GAPDH used as loading control.

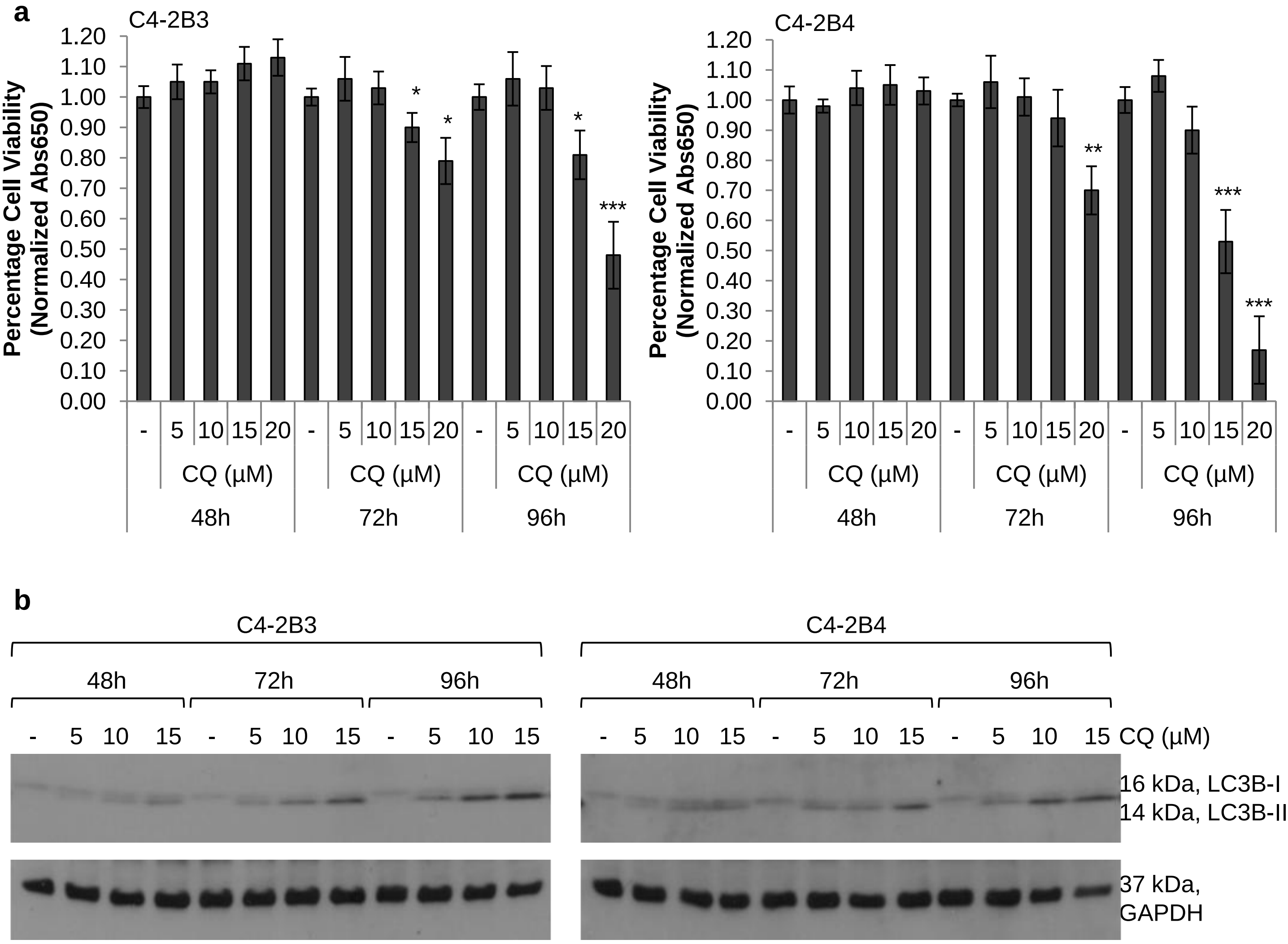

Supplementary Figure S3. 10µM chloroquine inhibits lysosomal function without significantly affecting C4-2B3 and C4-2B4 cell viability. (**a**) Percentage cell viability (mean ± S.D.) by methylene blue in C4-2B3 and C4-2B4 cells following treatment with 5-20 µM chloroquine (CQ) for 48-96h, n = 3 per group. * $P < 0.05$, ** $P < 0.01$, *** $P < 0.001$ determined by two-tailed Student's t test compared to respective untreated control. (**b**) Western blot analysis of LC3B protein expression from total cell lysates from C4-2B3 and C4-2B4 cells following treatment with 5-15 µM CQ for 48-96h. GAPDH used as loading control.

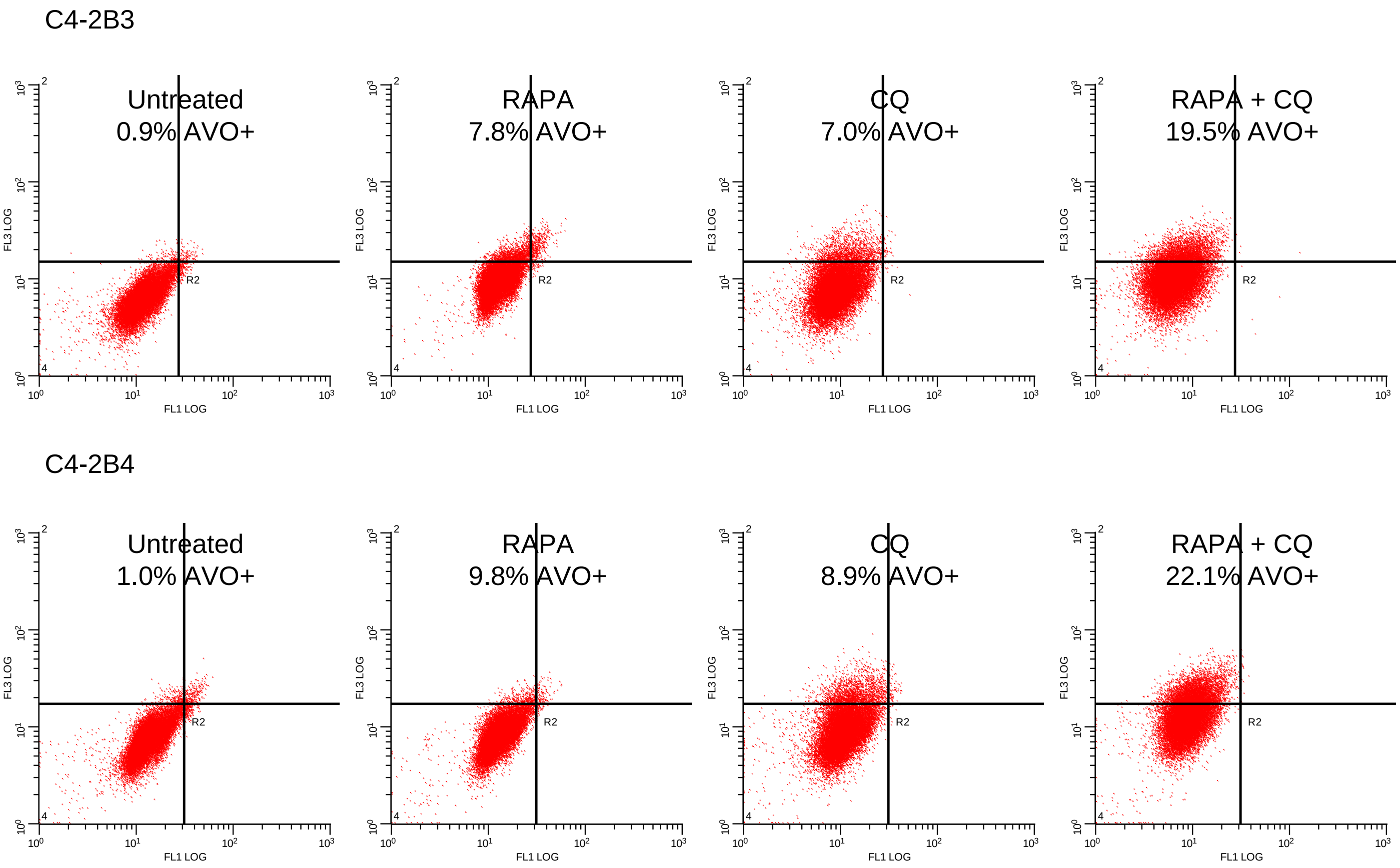

Supplementary Figure S4. Rapamycin positive controls for acridine orange flow cytometric quantification of acidic vesicular organelles. Representative density plots and quantification of acidic vesicular organelles (AVOs) by acridine orange staining using flow cytometry in C4-2B3 and C4-2B4 cells treated with 500 nM rapamycin (RAPA) ± 10 µM chloroquine (CQ) for 48h. In acridine orange-stained cells, the nucleaus fluoresces green (525 nm emission, x-axis), whereas acidic compartments fluoresce red (620 nm emission, y-axis). The intensity of the red fluorescence is proportional to the degree of acidity and to the volume of AVOs, including autophagic vacuoles. Mean (n = 2 per treatment) percentages of cells with a significant proportion of AVOs stated on plot.

Supplementary Table S1. Primary antibodies for immunoblotting and immunoprecipitation.

Primary Antibody	Manufacturer	Catalog No.
Total and cleaved caspase-3	Cell Signaling Technology	9662
Cleaved PARP (Asp214)	Cell Signaling Technology	9544
LC3B	Cell Signaling Technology	2775
Sequestosome 1 (SQSTM1/p62) (A-6)	Santa Cruz Biotechnology	sc-48402
Ripk1	BD Biosciences	610458
Ripk3	Enzo Life Sciences	ADI-905-242-100
HMGB-1	BD Biosciences	556528
Glyceraldehyde 3-phosphate dehydrogenase (GAPDH)	Cell Signaling Technology	2118
